# The effect of heat waves on pill bug *Armadillidium vulgare* risk-taking strategies

**DOI:** 10.1007/s00442-026-05968-5

**Published:** 2026-09-11

**Authors:** Sára Sarolta Sztruhala, Gergely Horváth, Gábor Herczeg

**Affiliations:** 1 Department of Systematic Zoology and Ecology, Institute of Biology, ELTE Eötvös Loránd University, Pázmány Péter Sétány 1/C, 1117 Budapest, Hungary; 2 HUN-REN-ELTE-MTM Integrative Ecology Research Group, Pázmány Péter Sétány 1/C, 1117 Budapest, Hungary; 3 Doctoral School of Biology, Institute of Biology, ELTE Eötvös Loránd University, Budapest, Hungary

**Keywords:** Behavioural type, Behavioural plasticity, Behavioural predictability, Climate change, Heat wave, Thermal stress

## Abstract

Global climate change is expected to increase both across- and within-day temperature fluctuations, likely affecting animal behaviour, particularly in small-bodied ectotherms. However, their impact on individual behavioural strategies remains understudied. Here, we investigated how heat waves influence risk-taking behaviour in the common pill bug *Armadillidium vulgare*, and whether prior experience with fluctuating temperatures alters this effect. Individuals were habituated to either fluctuating (alternating optimal 25ºC with stressful 31ºC daily) or stable (25ºC) thermal regimes. Risk-taking behaviour (conglobation time) was then repeatedly assessed at both 25ºC and 31ºC. Under thermal stress, *A. vulgare* exhibited reduced risk-taking, with 54–108% increase in conglobation time. This pattern was absent in females habituated to fluctuating thermal regime, showing only a minor decrease in risk-taking (~ 11%). Behavioural predictability (environment-independent within-individual behavioural variation) increased by 41% from the smallest to the largest individuals. Behavioural predictability decreased at higher temperatures in a sex-dependent fashion (females: 27%; males: 7%). Male repeatability was higher at 31ºC than at 25ºC in the fluctuating thermal treatment, while female repeatability was higher in the fluctuating than in the stable thermal treatment at 31ºC. Overall, our findings indicate that thermal stress alters risk-taking strategies and may promote between-individual behavioural differences, although prior thermal experience can buffer its effects. We conclude that climate change, even in the absence of other ecological changes, can reshape ectotherm antipredator strategies. We emphasize the need for long-term studies using the animal personality framework to understand how thermal landscape stability influences behavioural variation across ectotherm species.

**Supplementary Information:**

The online version contains supplementary material available at https://doi.org/10.1007/s00442-026-05968-5.

## Introduction

Consistent individual behavioural variation over time and/or across ecological situations (*animal personality*) is a commonplace phenomenon with various, important eco-evolutionary consequences (e.g. Sih et al. 2015; Niemelä and Dingemanse 2018; Mitchell et al. 2021; Moran et al. 2021). Besides individual *behavioural type* (i.e., individual differences in the mean value of a behavioural trait), studies started to focus on the within-individual components of behavioural variation. A dynamically growing body of empirical results indicates that consistent variation is present among individuals in their *behavioural plasticity* (i.e., the ability to adjust behavioural response to changing environmental conditions; Mathot et al. 2012, Johnston et al. 2019) and *behavioural predictability* (i.e., the precision of expressing the behavioural type under given environmental conditions; Biro and Adriaenssens 2013; Briffa 2013; Henriksen et al. 2020; Hertel et al. 2020, 2021). Empirical data suggest that apart from differences in average behaviour, variation in behavioural plasticity and predictability can be adaptive in several contexts, such as predator–prey interactions (Briffa 2013) and social niche partitioning (Bergmüller and Taborsky 2010).

The existence of animal personality differences remains a central research question, with several mutually non-exclusive explanations proposed. Differences in stable and labile features of individual state (e.g. size, sex, hormonal level, metabolism, parasite load) are often suggested to play a key role in the development and maintenance of consistent individual behavioural variation within population (Sih et al. 2015; Rádai et al. 2022), and this notion gained empirical support lately (DiRienzo et al. 2016; Horváth et al. 2017a, 2017b, 2020; Mathot et al. 2017; Niemelä and Dingemanse 2018; Royauté et al. 2019). Spatiotemporal differences in environmental conditions can affect the presence and strength of animal personality (e.g., Garamszegi et al. 2015; Horváth et al. 2017a, 2017b, 2020; Polverino et al. 2023; Reed et al. 2023; Hughes et al. 2025). Intuitively, under more fluctuating environmental conditions, the ability to be more plastic coupled with decreased predictability should provide advantages (see Herborn et al. 2014; Maskrey et al. 2020). Even though biotic and abiotic selective pressures vary spatially as well as temporally, far less focus has been given to the latter.

Temperature is perhaps the most important abiotic environmental factor that virtually influences every key aspect of life-history in ectotherms (i.e., animals whose internal temperature is primarily driven by environmental conditions) (Lee and Roh 2010; Rhen et al. 2011; Touchon and Warkentin 2011; Somero 2020). It obviously also affects animal personality (Biro et al. 2010; Briffa et al. 2013; Forsatkar et al. 2016; Abram et al. 2017). For instance, Biro et al. (2010) showed that even normal within-day fluctuations (< 3 C˚) of water temperature induced substantial changes in activity, boldness, and aggressiveness, altered behavioural ranks among individuals, indicating that temperature variability and climate change may increasingly disrupt behavioural patterns in ectotherms. Regarding within-individual behavioural variation, individual predictability appears to decrease at higher temperatures (Briffa et al. 2013; Maskrey et al. 2021), possibly driven by a trade-off between temperature-induced increases in metabolic demand and ectotherm predator activity. These results have far-reaching ecological and evolutionary implications, as extreme heat waves are predicted to increase in frequency, intensity and duration due to anthropogenic climate change (Meehl and Tebaldi 2004; Chiang et al. 2021), presenting many organisms a new range of challenges to cope with (Abrahams et al. 2007; Botero et al. 2015). However, experimental information regarding how stable *vs*. fluctuating temperature affects components of animal personality is rather sparse.

Our aim was to study how heat waves affect various components of personality of the common pill bug (*Armadillidium vulgare*), an important soil decomposer, and how prior experience with such waves shapes these responses. The preferred temperature range for *A. vulgare* is reportedly between 22–25°C, with an upper thermal tolerance around 36°C (cf. Edney 1964; Brody and Lawlor 1984; Refinetti 1984). We acclimated *A. vulgare* to either stable (25°C) or fluctuating (25°C and 31°C) thermal environments and repeatedly tested their risk-taking behaviour over six weeks at 25°C and 31°C. Here, 25°C is considered optimal and 31°C represents mild (unpreferred but tolerable) thermal stress. Our study design allowed us to examine how heat waves and habituation to heat waves, as well as differences in individual state (sex and size) influence behavioural type, plasticity, and predictability. We also examined the correlation structure among these components (cf. Horváth et al. 2023) and assessed the presence and strength of animal personality via behavioural repeatability. Given the complexity of our design, we do not form explicit predictions for all potential relationships. We generally hypothesized that mild heat stress induces a strong plastic response and results in increased between-individual behavioural divergence, reflected as higher repeatability (‘stronger’ personality). We further expected that prior experience with mild heat stress buffers this effect, potentially in a sex- and size-specific manner.

## Materials and methods

### Study species

*Armadillidium vulgare* (Latreille 1804) is a cosmopolitan terrestrial isopod (suborder: Oniscidea) with a broad environmental tolerance, which makes the species a widely used model organism in eco-evolutionary and physiological research (Csonka et al. 2013; Dittmer et al. 2016; Wenzel et al. 2018; Depeux et al. 2020; Prigot-Maurice et al. 2021). More recently, *A. vulgare* became an emerging subject of animal personality studies due to its typical defensive behaviour known as conglobation (see Horváth et al. 2019, 2025; Beveridge et al. 2022; Cornwell et al. 2023; Zamora-Camacho 2023). Pill bugs can roll their bodies into seamless sphere, during which they remain immobile. Conglobation serves as a defence strategy of pill bugs: it helps against visually oriented vertebrate predators by increasing their resemblance to the surrounding environment, while their robust tergites and dorsal sheets shield their vulnerable posterior appendages (uropods), legs and antennae from most invertebrate predators (Matsuno and Moriyama 2012; Tuf et al. 2015; Tuf and Ďurajková, 2022; Lough and Emberts 2026). However, the protective effectiveness and duration of the conglobation are state- and context-specific. For instance, smaller (and presumably younger) pill bugs have been shown to conglobate for longer periods, likely because predators more often disregard them when they are in an inactive state compared to larger, older individuals (Quadros et al. 2012).

Despite these variations, conglobation time serves as an excellent proxy of risk-taking behaviour, which can be used as an ecologically meaningful and objective measure of boldness (Horváth et al. 2019, 2025; Beveridge et al. 2022; Cornwell et al. 2023; Zamora-Camacho 2023). From a logistical perspective, conglobation time can be efficiently measured across multiple individuals within a short time frame. This allows for the collection of sufficient within-individual repeated measures (six repeats per individual and a total sample size > 120, i.e., number of repeats × number of individuals should provide adequate statistical power; see Horváth et al. 2023), thereby facilitating the use of current, data-intensive statistical approaches that would otherwise be difficult to apply in many other species.

### Collection and husbandry

We collected 80 *A. vulgare* (41 females, 39 males) on 10 October 2020 from a suburban garden located in Budapest, Hungary (47.519780˚ N, 19.148700˚ E; WGS 84). Strong sibling structure is our sample is unlikely, owing to the highly promiscuous mating system of *A. vulgare*: both males and females mate repeatedly (Moreau et al. 2002; Moreau and Rigaud 2003), and females commonly produce broods sired by multiple males (up to 5–7 fathers per brood; Valette et al. 2017). Consequently, sampling from one site does not imply non-independence due to shared parentage; rather, it reflects natural population structure.

Upon capture, animals were transported to the facilities of the Biological Institute of Eötvös Loránd University (Budapest, Hungary). Here, individuals were weighed using a digital scale to the nearest 0.01 g and housed separately in white plastic containers (18 cm × 13 cm × 5 cm, length × width × height, respectively) covered with a perforated semi-transparent lid, where they were kept during the whole time of capture. In the containers, a layer of mixed coconut peat and organic plant soil was used as substrate. As conglobation in terrestrial isopods also serves water conservation (Smigel and Gibbs 2008), soil moisture was maintained using a garden sprayer throughout the experiment. Animals were acclimated to a 12 h daily light cycle, controlled by a timer. Dim light was provided by Repti Glo 2.0 Full Spectrum Terrarium Lamps (Exo Terra, Rolf V. Hagen Inc., Holm, Germany), which do not emit considerable heat but mimic the full spectrum natural light. Food (slices of potatoes) was provided ad libitum. As it was shown in terrestrial isopods, including *A. vulgare*, a single copulation allows females to store enough sperm to fertilize several broods for more than one year (e.g., Suzuki 2001; Ziegler and Suzuki 2011), we decided to habituate the individuals for six months at room temperature (approximately 25 ºC, which is the higher end of the optimal temperature range; Edney 1964; Brody and Lawlor 1984; Refinetti 1984) before experiments to sort out females that may become gravid in the meantime.

### Experimental protocol

We created two experimental groups (N = 25 per group; individuals were selected randomly from the original pool, males and females were distributed evenly). In the fluctuating group, animals experienced daily changing temperature (25 C˚ *vs.* 31 C˚), while the stable group was kept on a constant room temperature of 25 C˚. We used 15W heat cables (ExoTerra, Hagen Inc., Montreal, QC) attached to a plastic board (83 cm × 56 cm, length × width) covered with black basalt sand to create a surface that was able to elevate the temperature of the soil in the boxes to 31 C˚ within 30 min. One round of the experiment consisted of six days of acclimation and a behavioural test on the seventh day. At the start of the acclimation period, half of the individuals (chosen randomly) in the fluctuating group were assigned to 31 C˚ temperature, while the other half to 25 C˚. Temperatures were switched (i.e., the position of the individual boxes was changed between surfaces) daily at 9.00 am (UTC + 02.00) in the fluctuating group, while at the same time, the position of individual boxes in the stable group was randomly rearranged on the shelves to standardize for the manipulation of that of the fluctuating group. Soil temperatures in the fluctuating and stable groups were regularly checked using a non-contact infrared thermometer (ST80 ProPlus™,Raynger®ST™, Raytek, Santa Cruz, CA, USA).

Individual behaviour was evaluated twice on the seventh day to obtain repeated measures needed for the repeated assessment of thermal plasticity. The first measurement took place at 9.30 am (UTC + 02.00) and the second at 4.00 pm (UTC + 02.00). The order of test temperatures (25 *vs.* 31 C˚) was randomised for the stable group, while individuals from the fluctuating group were first measured at the temperature they were kept at the day before and second at the other temperature. Stable group individuals measured at 31°C got placed at the heater at 9.00 am, and at the same time, the position of stable groups individuals measured at 25°C were randomly rearranged on the shelves. We estimated risk-taking by latency to restart activity (time spent immobile in conglobation) after a simulated predator attack (cf. Horváth et al. 2019). Basically, animals were removed from their home boxes using a tweezer, after which the experimenter gently squeezed the animal to trigger conglobation, then dropped it to the surface from a standard height of 10 cm. This treatment is similar to manipulation by larger vertebrate predators (e.g. birds and lizards) (see Tuf et al. 2015). Animals were considered to restart activity when they fully stretched their body and started to move their legs in an attempt to escape. If an individual did not restart activity in 5 min, the assay was stopped, and the individual was assigned a maximum score (300 s). This happened in 17% of all cases; here, we used the maximum score in the subsequent analyses. We conducted six rounds of measurements during consecutive 42 days, which translates to 12 behavioural scores per individual.

### Statistical analyses

Latency data were log-transformed to achieve normal distribution of model residuals. We ran double hierarchical generalized linear models (DHGLMs), using the R package brms (Bürkner 2017, 2018) based on the Bayesian software Stan (version 2.34.1, Carpenter et al. 2017; Stan Development Team 2024). A DHGLM is an extension of a random-intercept mixed model, and its main advantage over multi-step (stats-on-stats) approaches is that it allows for the simultaneous estimation of variation in both means and variances within a single overarching framework (see Cleasby et al. 2015 for details). In practice, a DHGLM simultaneously estimates a “mean model” and a “dispersion model.” The mean model estimates whether individuals differ in their mean expression of a behaviour (i.e., behavioural type), while the dispersion model estimates whether they differ in residual intra-individual variation (rIIV) around the behavioural mean (i.e., behavioural predictability). It is important to note, that population mean residual standard deviation (the intercept of the dispersion model) and individual specific residual standard deviation (i.e., rIIV) are estimated on the log scale to ensure that standard deviations are always positive (Cleasby et al. 2015). In our models, we also allowed the estimation of the correlation between the random intercepts in the mean model and dispersion model (i.e., the correlation between behavioural mean and rIIV) and the among-individual correlation of rIIVs.

The response variable (conglobation latency) was additionally standardized (mean = 0, SD = 1) and analysed using a Gaussian distribution; this transformation does not affect the parameters of interest but aids model fitting. The “mean model” part of our DHGLM was fitted by adding a population intercept, while trial days, body size, time of days (am *vs.* pm), treatment (stable *vs.* fluctuating), temperature (25 ºC *vs.* 31 ºC), sex, and the factorial interactions of the latter three were included as fixed effects. We fitted the variances for ID (i.e., random intercept) conditional to each treatment group. To test for temperature-induced plasticity, we fitted a random regression across temperature regimes, utilizing the so-called reaction norm approach (cf. Mitchell and Houslay 2021; Salazar et al. 2023; Kralj-Fišer et al. 2025). Random slopes (i.e., in interaction with individual identity), were fitted separately for each treatment group and we fitted temperature regime as a continuous covariate with re-coded conditions to − 0.5 (25 ºC) and 0.5 (31 ºC). The ‘dispersion model’ was fitted in a similar way: we modelled the residual standard deviation of the behavioural measure on the log scale, and the population intercept was controlled for the fixed effects of sex, treatment and temperature (and their factorial interaction). Random intercept of individual was also included, separately for each treatment group. Importantly, brms provides variance parameters in standard deviations, thus, these values must be squared to calculate the variance.

We ran four chains to evaluate convergence, which were run for 5,000 iterations, with a warmup of 1,000 iterations and a thinning interval of 4. All estimated model coefficients and intervals of posterior probability (i.e., credible intervals) were therefore based on 4,000 posterior samples and had satisfactory convergence diagnostics with Ȓ < 1.01 (compares the between- and within-chain estimates for model parameters and other univariate quantities of interest, see Bürkner 2017; Vehtari et al. 2020), and effective sample sizes >4,700 (see Vehtari et al. 2020).

To calculate adjusted repeatability for risk-taking of different sexes of treatment groups at different temperatures, we fitted two additional DHGLMs for females and males separately. In these models we utilized the so-called character state approach, an alternative parameterisation to the reaction norm approach by fitting an unstructured among-individual covariance matrix across temperature regimes. These two specifications are mathematically equivalent and can be re-parameterised into one another, but they offer complementary perspectives on environmental effects (cf. Mitchell and Houslay 2021; Salazar et al. 2023; Kralj-Fišer et al. 2025). In the character state framework, behaviour expressed at different temperatures is treated as distinct, but potentially correlated traits. This allows direct estimation of among-individual correlations in predicted mean behaviours across temperatures, thereby assessing whether individuals that behave in a certain way at one temperature tend to do so consistently at another. Importantly, this approach does not impose assumptions about the functional form of behavioural change across temperatures (e.g., linearity, as in reaction norm models) and thus permits a flexible evaluation of how risk-taking expressed in different thermal environments is related.

The character state DHGLMs were parametrized similarly to that described above (without ‘sex’ as an explanatory variable), but we fitted individual ID as a random intercept separately for each temperature regimes (fitted as a fixed factor with the levels 25 ºC *vs.* 31 ºC) and allowed this to differ between the two treatment groups to test for differences in behavioural variation between the temperature regimes. In the dispersion model, we allowed variance estimates to differ between the temperature regimes in each treatment groups to investigate how rIIV differed in different treatment groups across temperature regimes (cf. Beveridge et al. 2022; Brand et al. 2023). Adjusted repeatability of conglobation latency was calculated directly from these models by dividing variance explained by the among individual level (V_i_) by the total phenotypic variance occurs among- and within-individuals (V_i_ and V_w_, respectively).

$$R= {V}_{i}/({V}_{i}+{V}_{w})$$

We report posterior means and 85% credible intervals to visualise the central uncertainty of posterior expectations rather than to provide a dichotomous test of differences (cf. McElreath 2020). Inference about group differences is based on posterior contrasts (reported in the Results and ESM), not on interval overlap. All analyses were performed using R 4.5.1 (R Developmental Core Team 2025). Our R code and output are provided in the Open Science framework.

## Results

**Fig. 1:**
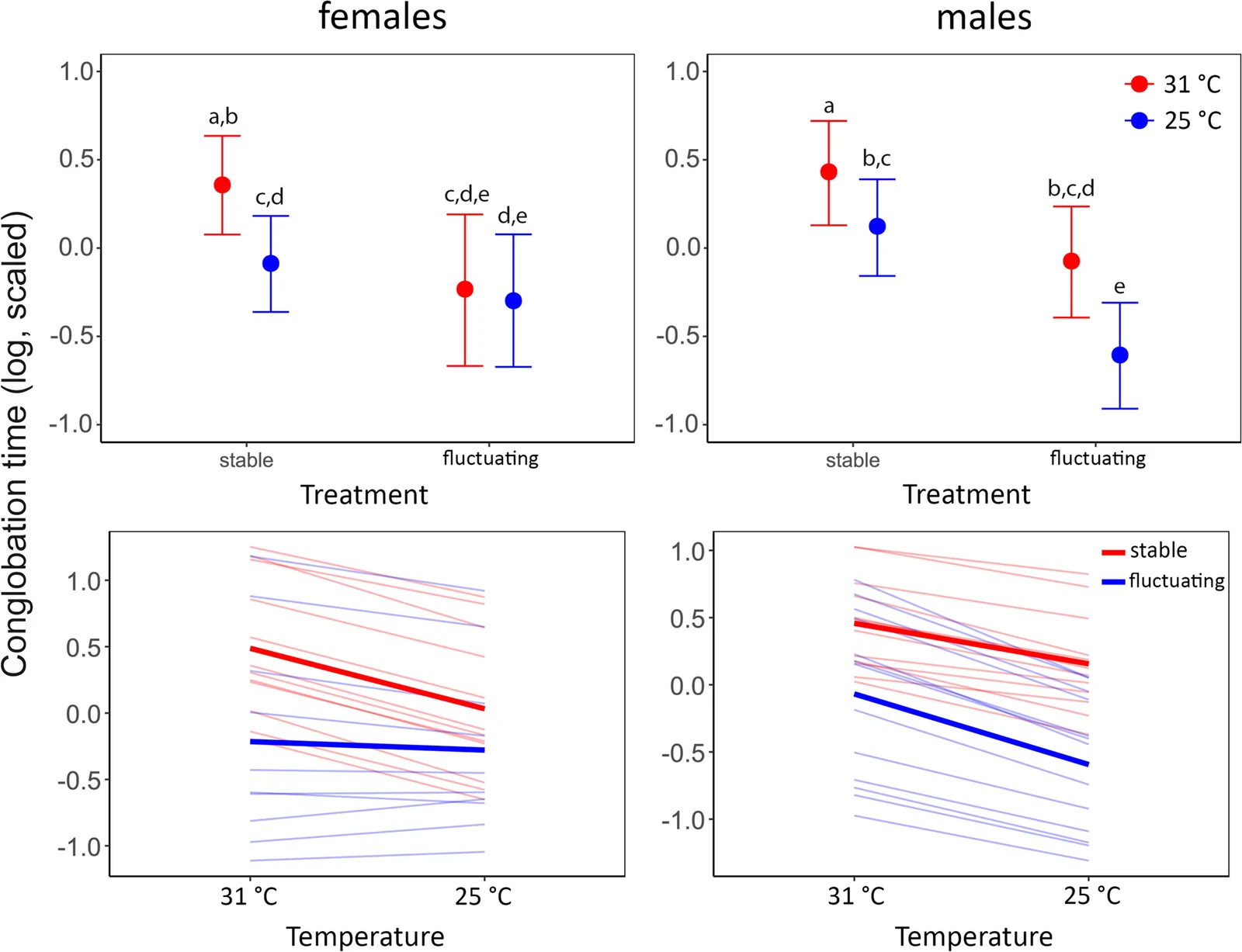
Population-level thermal plasticity (top row) and individual-level thermal plasticity (bottom row) of conglobation time in treatment groups habituated to stable or fluctuating environment in female and male *Armadillidium vulgare*. Population level plots represent conditional effects and 85% credible (posterior probability) intervals extracted from Bayesian double hierarchical mixed-effects models. Thin lines in individual-level plots represents the model predicted individual trendlines (reaction norms); thick lines indicate the mean level trendlines for females and males separately. Conglobation time is a latency variable; hence, lower values indicate higher risk-taking. Note that treatments marked with the same letter are not significantly different even between females and males (for further details see Supplementary Table S2)

Our DHGLM based on the reaction norm approach provided evidence for a three-way treatment × temperature × sex interaction on mean risk-taking (i.e., behavioural type) (Table 1). To facilitate biological interpretation of this interaction, we conducted two additional DHGLMs separately for females and males. These analyses showed that the treatment × temperature interaction had no effect in males, however, the main effects remained significant (Supplementary Table S1). Post-hoc comparisons based on the first DHGLM (see Supplementary Table S2) and posterior distribution of the difference between conditions (Fig. S1a) revealed a difference between temperatures in both treatment groups. Specifically, males were more risk-averse at 31 ºC (see Fig. 1), as indicated by longer conglobation durations, with a 54% increase in the stable group and a 108% increase in the fluctuating group (back-transformed posterior expectations are shown in Supplementary Table S3). In contrast, the treatment × temperature interaction was supported in females (Fig. 1, Supplementary Table S1). Post-hoc tests revealed that females in the stable group were more risk-averse at 31 ºC showing an 85% increase in conglobation time, whereas temperature had no effect in the fluctuating group (11% increase, Supplementary Table S2 and S3).

**Table 1:** Estimates and 85% credible (posterior probability) intervals (in parentheses) of fixed and random effects (mean model) and residual standard deviation (dispersion model) of conglobation time in *Armadillidium vulgare*

		Estimate (85% CI)
Mean model	*Fixed effects*	
	Intercept	**0.36 (0.09 – 0.63)**
	Time	**0.08 (0.04 – 0.13)**
	Size	**0.21 (0.08 – 0.35)**
	Treatment	
	Fluctuating	**-0.62 (− 1.06 – − 0.18)**
	Temperature	
	25°C	**-0.45 (− 0.65 – − 0.24)**
	Sex	
	Male	0.04 (− 0.31 – 0.41)
	Time of day	
	pm	0.09 (− 0.005 – 0.18)
	Treatment × temperature	**0.38 (0.08 – 0.68)**
	Treatment × sex	0.18 (− 0.41 – 0.76)
	Temperature × sex	0.13 (− 0.16 – 0.43)
	Treatment × temperature × sex	**-0.59 (− 1.02 – − 0.18)**
	*Random effects*	
	Stable treatment	
	sd_intercept.ID_	**0.51 (0.38 – 0.66)**
	sd_slope.temperature_	**0.18 (0.02 – 0.38)**
	*r*_intercept.ID-slope.temperature_	-0.07 (− 0.69 – 0.59)
	*r*_intercept.ID-σ.ID_	-0.37 (− 0.85 – 0.25)
	Fluctuating treatment	
	sd_intercept.ID_	**0.69 (0.52 – 0.89)**
	sd_slope.temperature_	**0.2 (0.04 – 0.38)**
	*r*_intercept.ID-slope.temperature_	-0.57 (− 0.93 – 0.05)
	*r*_intercept.ID-σ.ID_	0.1 (− 0.55 – 0.72)
Dispersion model	*Fixed effects*	
	Intercept	**-0.17 (− 0.31 – − 0.03)**
	Size	**-0.11 (− 0.17 – − 0.05)**
	Treatment	
	fluctuating	-0.14 (− 0.35 – 0.07)
	Temperature	
	25°C	**-0.22 (− 0.41 – − 0.04)**
	Sex	
	male	-0.08 (− 0.28 – 0.12)
	Treatment × temperature	0.12 (− 0.16 – 0.4)
	Treatment × sex	0.02 (− 0.26 – 0.32)
	Temperature × sex	0.16 (− 0.11 – 0.43)
	Treatment × temperature × sex	0.09 (− 0.29 – 0.48)
	*Random effects*	
	stable treatment	
	σ.ID	**0.12 (0.03 – 0.22)**
	*r*_slope.temperature-σ.ID_	-0.13 (− 0.79 – 0.62)
	fluctuating treatment	
	σ.ID	**0.11 (0.02 – 0.22)**
	*r*_slope.temperature-σ.ID_	0.01 (− 0.69 – 0.72)

Estimates are based on double hierarchical mixed models (reaction norm method). Estimates which were different from 0 based on their 85% credible intervals are bolded (note: variance estimates cannot overlap with zero as they are positively bound). Females in the stable group at 31°C are set as reference group

However, in the fluctuating group, females exhibited a relatively weak mean plastic response to temperature differences (Fig. 1), indicated also by back-transformed posterior expectations (Supplementary Table S3 and Fig. S1a). This pattern is driven by the variation in both the slope and elevation of individual reaction norms within the fluctuating treatment (Fig. 1), compared to males.

**Fig. 2:**
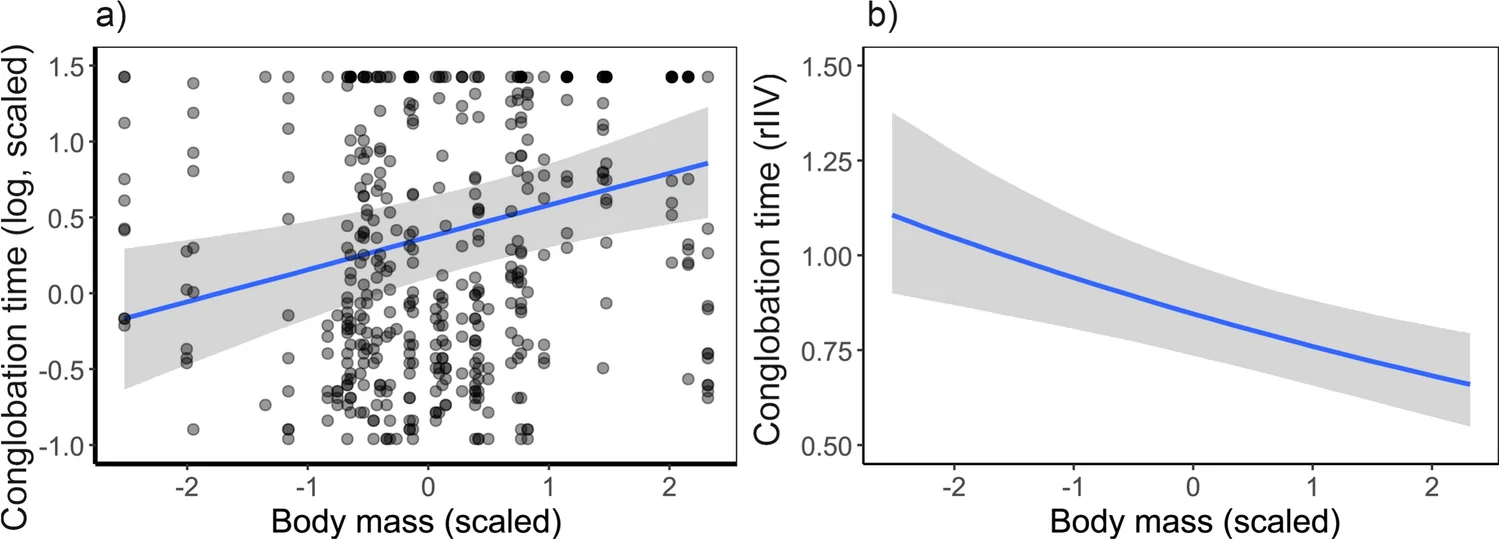
Size (body mass) effect on population-level a) mean conglobation time (i.e., behavioural type) and b) residual intra-individual variation (rIIV) of conglobation time (i.e., behavioural predictability) in *Armadillidium vulgare*. Shaded areas represent 85% credible (posterior probability) intervals. Conglobation time is a latency variable; hence, lower values indicate higher risk-taking

**Fig. 3:**
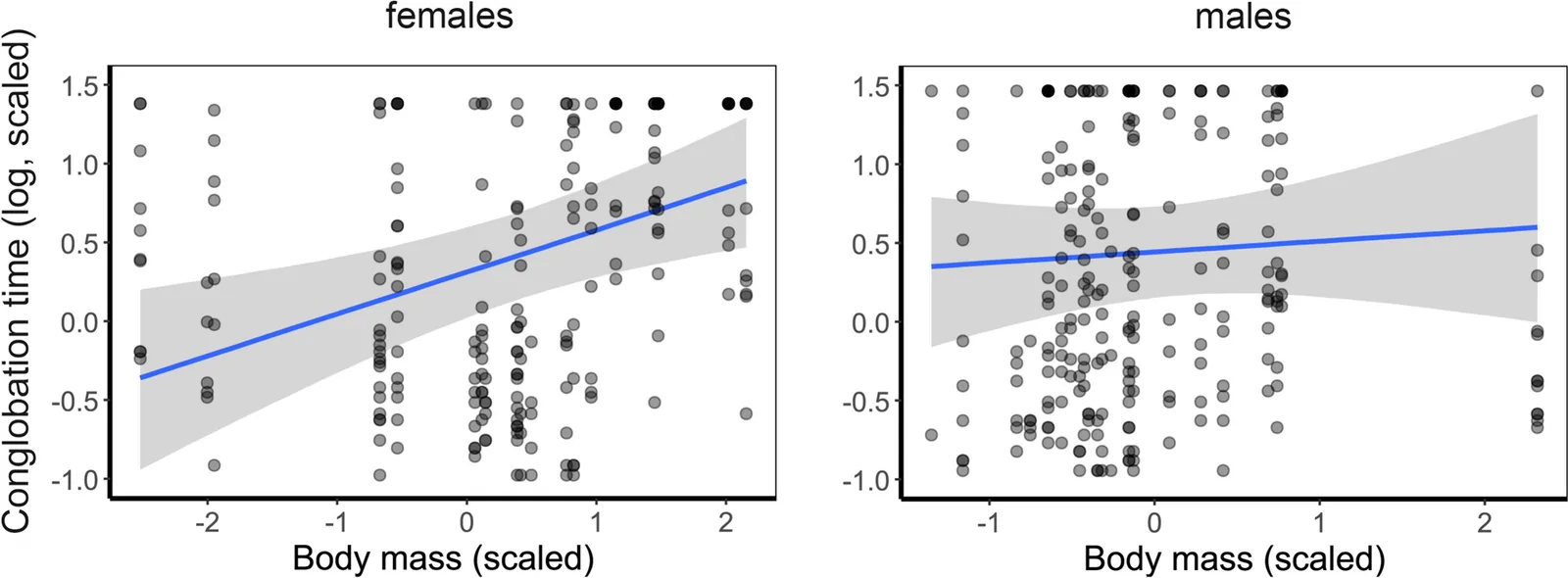
Size (body mass) effect on population-level mean conglobation time (i.e., behavioural type) in females and males of *Armadillidium vulgare*. Shaded areas represent 85% credible (posterior probability) intervals. Conglobation time is a latency variable; hence, lower values indicate higher risk-taking

**Fig. 4:**
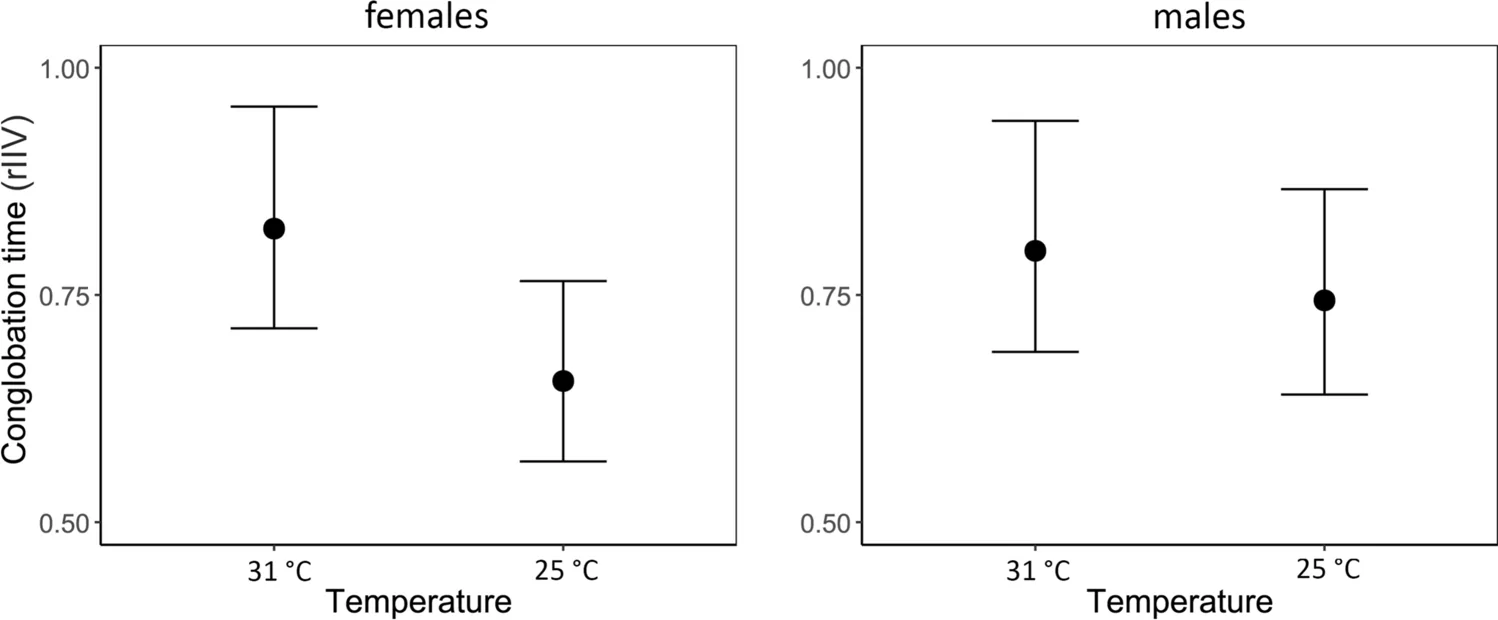
Temperature effect on residual intra-individual variation (rIIV) of conglobation time (i.e., behavioural predictability) in females and males of *Armadillidium vulgare*. Plots represent conditional effects, and 85% credible (posterior probability) intervals extracted from Bayesian double hierarchical mixed-effects models. The difference is significant for females, but not for males

We detected significant between-individual variation in risk-taking behavioural type (sd_intercept.ID_), plasticity (sd_slope.temperature_) and predictability (sd_σ.ID_) in both treatments (Table 1). Size (i.e., body mass) influenced mean risk-taking (i.e., behavioural type) (Table 1). Back-transformed posterior expectations showed that expected conglobation duration increased from 24.65 s at the minimum observed body mass to 101.34 s at the maximum observed body mass, corresponding to an approximately fourfold increase and indicating reduced risk-taking in larger individuals. (Fig. 2a). However, DHGLMs run separately for each sex revealed that this effect was driven by females (see Supplementary Table S1, Fig. 3). Additionally, size affected predictability (Table 1). Smaller *A. vulgare* were generally less predictable (exhibited higher rIIV) than larger ones (Fig. 2b). Back-transformed posterior expectations indicated that residual variation decreased from 3.55 s at the minimum observed body mass to 2.10 s at the maximum observed body mass, corresponding to ~ 41% reduction and thus higher behavioural predictability in larger individuals. Temperature differences also influenced risk-taking predictability (Table 1), with *A. vulgare* being less predictable at 31 ºC, but the overall effect, again, was driven by females (Supplementary Table S1 and Fig. S1b): decrease being 27% in females and 7% in males (Fig. 4; Supplementary Table S3). Finally, we observed a weak trend of *A. vulgare*, irrespective of sex, becoming shier over time (Table 1), suggesting a sensitization effect (Fig S2).

We detected no correlation between behavioural type (sd_intercept.ID_), behavioural predictability (sd_σ.ID_), and behavioural plasticity (sd_slope.temperature_) in either treatment group (see Table 1). These results remained consistent when the sexes were analysed separately in the reaction norm approach (see Supplementary Table S1). However, our character state DHGLMs revealed positive across-temperature correlations in behavioural type in the fluctuating treatment group for both sexes, but this correlation was present only in females from the stable treatment group (Supplementary Table S4). Character state DHGLMs also revealed no correlations between behavioural type and behavioural predictability across temperatures, nor between behavioural predictability estimates across temperatures, regardless of treatment groups or sex (Supplementary Table S4).

Repeatability estimates indicate that both female and male *A. vulgare* exhibit risk-taking personality (females: R = 0.35 [0.2 – 0.47]; males: R = 0.32 [0.16 – 0.46]). Estimates for *A. vulgare* in the stable and fluctuating treatment groups revealed no statistically detectable differences (stable: R = 0.26 [0.15 – 0.37]; fluctuating: R = 0.42 [0.27 – 0.56]). We tested for repeatability differences between different treatment groups at 25 ºC and 31 ºC separately, as well as differences between temperatures within treatment groups (Table 2). Our results revealed sex-specific differences in repeatability. In males of the fluctuating group, there was strong posterior support for repeatability difference between temperatures: *A. vulgare* males were almost two times more repeatable at 31 ºC than at 25 ºC (see Supplementary Table S5; Fig S3a). No such difference was found in the stable group, or between treatment groups. In females, there was a strong posterior support for difference between stable and fluctuating groups at 31 ºC: *A. vulgare* females in the fluctuating group exhibited almost three times higher repeatability than females in the stable group (Supplementary Table S5; Fig S3b). No such difference was observed at 25 ºC, or between temperatures. Furthermore, we found strong posterior support for repeatability differences between the sexes across treatment groups and temperatures in two cases: fluctuating females at 31°C were twice as repeatable as stable males at 25°C; additionally, fluctuating males were twice as repeatable as stable females at 31°C (Supplementary Table S5).

**Table 2:** Between (V_i_)- and within-individual variance (V_w_) and repeatability (R) estimates (85% credible [posterior probability] intervals) for conglobation time of female and male *Armadillidium vulgare* in the treatment groups at both 25°C and 31°C

			V_i_ mean (85% CI)	V_w_ mean (85% CI)	R mean (85% CI)
*Sex*	*Treatment*	*Temperature*			
Female	Stable	25°C	0.33 (0.08 – 0.53)	0.45 (0.29 – 0.59)	0.4 (0.19 – 0.6)
		31°C	0.2 (0.01 – 0.33)	0.72 (0.46 – 0.96)	0.2 (0.03 – 0.34)
	Fluctuating	25°C	0.41 (0.06 – 0.66)	0.45 (0.26 – 0.6)	0.44 (0.19 – 0.68)
		31°C	0.82 (0.2 – 1.28)	0.54 (0.35 – 0.71)	0.57 (0.37 – 0.78)
Male	Dtable	25°C	0.26 (4.32 × 10^–5^ – 0.43)	0.54 (0.34 – 0.7)	0.27 (0.06 – 0.47)
		31°C	0.43 (0.06 – 0.7)	0.62 (0.43 – 0.79)	0.38 (0.16 – 0.59)
	fluctuating	25°C	0.27 (0.03 – 0.46)	0.68 (0.44 – 0.9)	0.27 (0.07 – 0.45)
		31°C	0.56 (0.15 – 0.87)	0.5 (0.33 – 0.66)	0.49 (0.31 – 0.71)

## Discussion

One of the most important aspects of ongoing global climate change is the increasing frequency of extreme temperature fluctuations, both across and within days, seasons and years (Meehl and Tebaldi 2004; Chiang et al. 2021). To cope with fluctuations in ambient temperature, small-bodied ectotherms rely on various physiological and behavioural adaptations, as their body temperature quickly equilibrates with that of the surrounding environment. Further, such environmental extremes might induce plasticity in traits unrelated to coping, resulting in maladaptive changes. Here we studied how simulated heat waves affect risk-taking strategy of experienced *vs*. naïve *A. vulgare*.

### The effects of environmental variation on animal personality

Adjusted repeatability estimates indicated the presence of animal personality in both treatment groups and at both temperatures. Repeatability ranged from 0.2 to 0.57, corresponding to moderate to high values (global mean = 0.37; Bell et al. 2009). Higher repeatability reflects stronger among-individual differentiation, that is, more distinct individual behavioural strategies. The highest estimates are coming from the fluctuating group recorded at 31°C, while the lowest estimates are recorded in the stable group, although at different temperatures (females at 31°C, males at 25°C). Our estimates are comparable with what is known about risk-taking repeatability in *A. vulgare* (Cornwell et al. 2023; Horváth et al. 2025).

The detected repeatability estimates suggest an increase in repeatability as a response to heat wave, except in stable treatment females. However, based on the 85% CIs, only males from the fluctuating treatment showed a strong posterior support for repeatability increase between 25°C and 31°C (cf. Royauté and Dochtermann 2021). There is a numerical trend for higher repeatabilities in the fluctuating than in the stable treatment in females, however, the difference was statistically meaningful only at 31°C. Given that 31°C is way above *A. vulgare*’s thermal preference range, but below the upper boundary of *A. vulgare*’s thermal tolerance range (cf. Edney 1964; Refinetti 1984), this temperature likely constitutes a mildly stressful, i.e., not lethal, environmental challenge. Hence, our results suggest that mild environmental stress has a diversifying effect on individual behavioural strategies. Further, we found some evidence that environmental fluctuation can also diversify individual behavioural strategies under challenging conditions, which is in line with our previous findings in another model system (Urszán et al. 2015, 2018).

An open question remains as to whether the emergence of animal personality is more strongly favoured under optimal or suboptimal (i.e., stressful) environmental conditions (e.g., Careau et al. 2014; Urszán et al. 2015, 2018; Wexler and Scharf 2017; Horváth et al. 2017a, 2017b; Grunst and Grunst 2024). To date, research has primarily focused on biotic factors, such as the presence or absence of predators, predator cues, conspecific density, or variations in food availability, rather than abiotic stressors (e.g., Toscano et al. 2014; Urszán et al. 2015, 2018; Horváth et al. 2017b; Segovia et al. 2019; Scherer et al. 2024). Moreover, most studies fail to distinguish between mild and severe stress. Mild stress occurs when environmental conditions deviate from the optimal range but remain within tolerable, non-lethal thresholds. In contrast, severe stress pushes organisms close to or beyond their tolerance limits, creating life-threatening conditions. Our results support the idea that mild stress should promote behavioural diversification, as individuals can adopt multiple coping strategies, or the stress itself can affect individuals of different state differently. Conversely, severe stress is likely to canalize behavioural responses, as survival may depend on a single, viable strategy, however, this hypothesis warrants dedicated studies that examine various stress-levels simultaneously.

### Differences in behavioural type and plasticity

Importantly, contrary to stable group females who became almost twice as risk-averse at 31°C (back-transformed posterior expectations of conglobation time are 37.2 s at 25°C *vs.* 69 s at 31°C), fluctuating group females did not exhibit considerable population-level temperature-induced behavioural plasticity (back-transformed posterior expectations of conglobation time are 26.9 s at 25°C *vs.* 30 s at 31°C). Males became more risk-averse at 31°C regardless of treatment group (i.e., back-transformed posterior expectations of conglobation time in the stable group are 47.3 s at 25°C *vs.* 72.9 s at 31°C, and in the fluctuating group are 19.2 s *vs.* 40.1 s).

As noted by Urszán et al. (2018), population-level plasticity (i.e., population-level reaction norms) cannot be fully understood without considering individual reaction norms. For instance, an apparent lack of population-level plasticity may be due to i) individuals in the group exhibiting no plasticity, or ii) high variation in plasticity among individuals, with opposite directions. In our study, population-level patterns suggest that female *A. vulgare* in the fluctuating temperature group became desensitized to the unfavourable high temperature. On the individual level, stable group females and all males generally reduced their risk-taking in response to rising temperature. Females in the fluctuating group either showed a lower decrease or did not respond at all. Building on Edney's (1964) findings, which demonstrated that long-term exposure to alternating temperatures (12 h at 10°C and 12 h at 30°C for 14 days) expanded the voluntarily selected temperature range in *A. vulgare*, we can infer that even brief prior experience with 31°C was sufficient for females in the fluctuating group not to alter their risk-taking behaviour under mildly stressful temperatures.

But why did not males in the fluctuating group react in the same way as females? Sex differences in animal personality are well-documented across both vertebrate and invertebrate taxa (Harrison et al. 2022), and thermal behavioural plasticity has also been observed to differ among sexes, particularly in *Drosophila* flies (Latimer et al. 2011; Alton et al. 2020; Brand et al. 2023). However, previous studies suggest no sex-specific differences in thermal preference for *A. vulgare* (cf. Edney 1964). One possibility is that fluctuating group males did not become desensitized; in other words, 31°C might have been still stressful for them.

### The effects of individual state on behavioural type and predictability

We found that larger *A. vulgare* was more risk-averse than smaller conspecifics. However, when analysed separately, this effect was only evident in females, but not in males. Although the risk-taking level of males was not higher than that of females, it was at the higher end of female risk-taking (see Fig. 3). Large size variability exists in both female and male *A. vulgare* (see Lefebvre et al. 2019), but no sexual size dimorphism is reported, which is supported by our own observations as well. Regarding females, as *A. vulgare* is at least partially iteroparous (i.e., capable of producing multiple broods over its lifetime; cf. Dangerfield and Hassal 1992; Warburg 1993), size is a key determinant of mating outcome and it positively correlates with fertility and fecundity—larger females produce more eggs (Lefebvre et al. 2019) and are expected to produce more broods per season (Lawlor 1976). Given this, size functions as a state variable that should influence female behaviour. However, no size-dependent effect on risk-taking personality has been previously reported in this species (cf. Horváth et al. 2019; Beveridge et al. 2022; Cornwell et al. 2023). Most plausibly larger females exhibit lower risk-taking to avoid visually driven predators and to secure their high expected future reproductive success, consistent with the asset protection principle (Clark 1994; Dall et al. 2004; Wolf et al. 2007; Harcourt et al. 2009; Luttbeg and Sih 2010; Dosmann et al. 2014; Engqvist et al. 2015; Horváth et al. 2017b).

Larger *A. vulgare* individuals were found to be more predictable, regardless of sex. The antipredator value of unpredictability is often discussed: since predators tend to exploit repetitive prey behaviour, unpredictability may serve as an adaptive strategy to reduce the risk of capture (cf. Hugie 2003, Jones et al. 2011, Richardson et al. 2018, Horváth et al. 2019, but see Urszán et al. 2018). In the case of *A. vulgare*, large size might be a disadvantage when facing visually oriented vertebrate predators (cf. Tuf and Ďurajková 2022). For such animals, a risk-averse behavioural strategy—characterized by long conglobation times and low within-individual variation (i.e., high predictability)—may be most advantageous.

*Armadillidium vulgare* was more predictable at 25°C than at 31°C. However, when analysed separately, this effect was only evident in females. As part of the aforementioned antipredator strategy, increased behavioural variability may further reduce capture risk (Hugie 2003; Jones et al. 2011; Richardson et al. 2018; Horváth et al. 2019), particularly when ectothermic predators are more active at higher temperatures. Of course, besides this possible antipredator strategy (adaptive explanation), we cannot exclude the possibility that some direct physiological constraints are responsible for the observed increase in female rIIV (constraint explanation). Additionally, considering that among-individual behavioural differences emerge from either (i) divergence in mean behaviour or (ii) decrease in rIIV, it is important to note that repeatability estimates tended to be higher at 31°C (with the exception of stable females). High repeatability coupled with low predictability (i.e., high rIIV) indicates substantial divergence in behavioural types. This finding is again consistent with our hypothesis regarding the diversifying effect of mild stress.

Finally, *A. vulgare* individuals regardless of treatment or sex became slightly shier over time, indicating the presence of sensitization—an internal mechanism that amplifies behavioural responses to repeated stimulation (Bee 2001; Martin and Réale 2008; Stamps et al. 2012; Osborn and Briffa 2017). This phenomenon has been previously documented in *A. vulgare* (Horváth et al. 2019; Beveridge et al. 2022). However, compared to these earlier studies, particularly Horváth et al. (2019), the level of sensitization observed here was relatively small. This aligns with the lower intensity of our assays (i.e., once a week during six weeks in the present study *vs.* 30 times in 38 days [Horváth et al. 2019] and 24 times in three weeks [Beveridge et al. 2022]), as higher levels of sensitization are generally associated with more frequent or intense stimuli (Bee 2001).

### Correlations between behavioural type, predictability and plasticity

There was strong evidence of among-individual differences not only in risk-taking behavioural type but also in its plasticity and predictability. This suggests that *A. vulgare* vary not just in their behavioural mean but also in the degree of behavioural change across temperatures and the consistency with which they express risk-taking. This aligns with growing empirical evidence that behavioural plasticity and predictability are key components of an individual's behavioural strategy and potential targets of natural selection (see Hertel et al. 2021; O’Dea et al. 2022; Horváth et al. 2023, 2024). Estimates for individual variation in plasticity and predictability between the stable and fluctuating groups (respectively) were nearly identical, as well as between sexes (for treatment × sex estimates in plasticity [sd_slope.temperature_] see Supplementary Table S2, for treatment × temperature × sex estimates in predictability [sd_σ.ID_] see Supplementary Table S3), indicating that different acclimation regimes did not affect within-individual behavioural variation.

No correlations were found between behavioural type and plasticity, behavioural type and predictability, or between plasticity and predictability in any treatment group or sex. This suggests that neither behavioural plasticity nor predictability is linked to behavioural type. The absence of behavioural type–predictability correlations is somewhat surprising given recent meta-analyses by Mitchell et al. (2021) and Horváth et al. (2023), which report these links as relatively common. However, Horváth et al. (2023) also highlight that such correlations tend to be highly heterogeneous and system-specific. Our findings suggest that risk-taking behavioural type, plasticity, and predictability may evolve independently in the studied *A. vulgare* population, even if increasing ambient temperatures are considered as a selection factor.

Among-individual correlations in risk-taking across temperatures were present in both sexes, except for males in the stable group. This indicates re-ranking in stable males (cf. Brommer 2013), meaning that although individuals differ in their risk-taking behaviour (i.e., there are significant among-individual differences), the rank order of these values changes across environmental conditions—in this case, different temperatures. Such patterns have been rarely tested, and recent studies report little evidence for widespread re-ranking across temperatures (e.g., *Drosophila melanogaster*, Brand et al. 2023; *Cepaea nemoralis*, Dahirel et al. 2021). Nevertheless, in his seminal paper, Brommer (2013) reported generally low cross-environmental correlations (~ 0.4). In our study, when correlations were detected, they were relatively high (0.63–0.70), indicating that the rank order of among-individual differences remained largely consistent across temperature treatments. However, our findings on stable males suggest that risk-prone individuals do not necessarily remain risk-prone when exposed to changing temperature regimes. This is important for several reasons. First, if individual rankings change across environments, then selection operating in one environment may not predict evolutionary responses in another. This limits the stability of adaptive evolution and suggests that personality traits may have context-dependent fitness consequences (see Dingemanse et al. 2010). Second, rank-order changes can contribute to the maintenance of behavioural variation within populations; if no single behavioural type performs best across all situations, selection may favour the maintenance of diversity within the population. Finally, from a methodological perspective, re-ranking is important to consider, as it implies that behavioural measures obtained in one context may fail to predict behaviour in other contexts.

## Conclusion

In summary, our findings show that both thermal fluctuations and ambient temperature influence risk-taking in *A. vulgare* across multiple levels of behavioural variation. Importantly, the divergence of individual behavioural strategies increased at a mildly stressful temperature (31°C), but this depended on both the stability of the thermal landscape and sex. This suggests that increasing temperatures might open the studied population for stronger predator-imposed natural selection when they are under mild thermal stress. We found strong evidence of individual differences in behavioural type, plasticity, and predictability, regardless of treatments or sex, but without correlations. This implies that these components might evolve independently, irrespective of temperature regime. *A. vulgare* took less risk and was less predictable – which can also be effective strategy against predators – at 31°C. This might be an adaptive response against ectotherm predators performing best at this temperature, especially considering the lower thermal preferences of *A. vulgare* (Edney 1964; Brody and Lawlor 1984; Refinetti 1984) and the proven lower thermal optima for locomotor performance in the closely related *Porcellio laevis* (Castañeda et al. 2004). Alternatively, it can be a byproduct of lowered physiological performance of *A. vulgare* itself. Sex-specific differences in behavioural variation in response to temperature shifts and thermal landscape fluctuations may lead to sex-specific vulnerabilities in *A. vulgare* facing climate change. Given that ectothermic species are particularly responsive to temperature variation and environmental change (Custer et al. 2024; Paaijmans et al. 2013; Wagner et al. 2023), long-term studies applying advanced animal personality methodologies will be essential to clarify how thermal landscape dynamics influence behavioural variation and, by extension, ecological and evolutionary processes in ectothermic species.

## Supplementary Information

Below is the link to the electronic supplementary material.

Supplementary file1 (DOCX 770 KB)

## Data Availability

Data and detailed code for the analyses can be found at the Open Science Framework (OSF): https://osf.io/aqgtz/?view_only=168a6d0aac5941928b2d02303ec88a38.
