# Supplementary material for: The effect of heat waves on pill bug Armadillidium vulgare risk-taking strategies
Source: Oecologia. 2026 Sep 11;208(10):133. doi: 10.1007/s00442-026-05968-5 (PMC13569582; doi:10.1007/s00442-026-05968-5)
Supplement: Supplementary file 1 [file 442_2026_5968_MOESM1_ESM.docx]

Supplementary Table S1. Estimates and 85% credible (posterior probability) intervals (in parentheses) of fixed and random effects (mean model) and residual standard deviation (dispersion model) of conglobation time in female and male *Armadillidium vulgare*. Estimates are based on double hierarchical mixed models (reaction norm method). Estimates which were different from 0 based on their 85% credible intervals are bolded (note: variance estimates cannot overlap with zero as they are positively bound). *A. vulgare* in the stable group at 31 °C are set as reference group.

|  | model | |  | estimate (85% CI) | |
| --- | --- | --- | --- | --- | --- |
|  |  | |  | female | male |
|  | *Mean model* | | *Fixed effects* |  |  |
|  |  | | Intercept | **0.39 (0.02 – 0.59)** | **0.44 (0.15 – 0.73)** |
|  |  | | Time | **0.08 (0.02 – 0.14)** | **0.09 (0.03 – 0.16)** |
|  |  | | Size | **0.27 (0.09 – 0.44)** | 0.08 (-0.17 – 0.36) |
|  |  | | Treatment |  |  |
|  |  | | fluctuating | **-0.59 (-1.11 – -0.08)** | **-0.5 (-0.94 – -0.07)** |
|  |  | | Temperature |  |  |
|  |  | | 25 °C | **-0.45 (-0.64 – -0.25)** | **-0.31 (-0.57 – -0.05)** |
|  |  | | Time of day |  |  |
|  |  | | pm | 0.09 (-0.033 – 0.22) | 0.09 (-0.03 – 0.23) |
|  |  | | Treatment × temperature | **0.38 (0.06 – 0.7)** | -0.22 (-0.56 – 0.12) |
|  |  | | *Random effects* |  |  |
|  |  | | stable treatment |  |  |
|  |  | | sd_intercept.ID_ | **0.54 (0.35 – 0.78)** | **0.51 (0.3 – 0.78)** |
|  |  | | sd_slope.temperature_ | **0.17 (0.02 – 0.38)** | **0.39 (0.08 – 0.75)** |
|  |  | | *r*_intercept.ID-slope.temperature_ | -0.16 (-0.8 – 0.6) | -0.15 (-0.48 – 0.72) |
|  |  | | *r*_intercept.ID-σ.ID_ | -0.38 (-0.89 – 0.34) | -0.08 (-0.73 – 0.62) |
|  |  | | fluctuating treatment |  |  |
|  |  | | sd_intercept.ID_ | **0.8 (0.51 – 1.19)** | **0.66 (0.44 – 0.94)** |
|  |  | | sd_slope.temperature_ | **0.3 (0.05 – 0.61)** | **0.21 (0.03 – 0.45)** |
|  |  | | *r*_intercept.ID-slope.temperature_ | 0.36 (-0.35 – 0.88) | 0.29 (-0.47 – 0.87) |
|  |  | | *r*_intercept.ID-σ.ID_ | -0.02 (-0.66 – 0.62) | 0.18 (-0.56 – 0.81) |
|  | Dispersion model | | *Fixed effects* |  |  |
|  | | Intercept | | **-0.17 (-0.32 – -0.02)** | **-0.23 (-0.39 – -0.08)** |
|  | | Size | | **-0.1 (-0.18 – -0.03)** | **-0.12 (-0.23 – -0.001)** |
|  | | Treatment | |  |  |
|  | | fluctuating | | -0.15 (-0.39 – 0.09) | -0.11 (-0.33 – 0.1) |
|  | | Temperature | |  |  |
|  | | 25 °C | | **-0.23 (-0.41 – -0.04)** | -0.07 (-0.27 – 0.12) |
|  | | Treatment × temperature | | 0.12 (-0.16 – 0.41) | 0.23 (-0.04 – 0.49) |
|  |  | | *Random effects* |  |  |
|  |  | | stable treatment |  |  |
|  |  | | σ.ID | **0.15 (0.02 – 0.29)** | **0.14 (0.021 – 0.29)** |
|  |  | | r_slope.temperature-σ.ID_ | 0.09 (-0.67 – 0.79) | -0.3 (-0.88 – 0.46) |
|  |  | | fluctuating treatment |  |  |
|  |  | | σ.ID | **0.21 (0.03 – 0.42)** | 0.1 **(0.02 – 0.23)** |
|  |  | | r_slope.temperature-σ.ID_ | 0.01 (-0.71 – 0.72) | 0.02 (-0.73 – 0.76) |

Supplementary Table S2. Between-group differences (grouping factors including treatment, temperature, and sex) in conglobation time type of *Armadillidium vulgare* revealed by post-hoc Tukey tests. Estimates are based on a single double hierarchical generalized mixed model based on the reaction norm approach. Estimates which were different from 0 based on their 85% highest posterior density (HPD) intervals are bolded.

| contrast | estimate (85% HPD) |
| --- | --- |
| stable female 31 °C – fluctuating female 31 °C | **0.62 (0.18 – 1.06)** |
| stable female 31 °C – stable female 25 °C | **0.44 (0.24 – 0.64)** |
| stable female 31 °C – fluctuating female 25 °C | **0.69 (0.29 – 1.08)** |
| stable female 31 °C – stable male 31 °C | -0.04 (-0.39 – 0.33) |
| stable female 31 °C – fluctuating male 31 °C | 0.39 (-0.01 – 0.82) |
| stable female 31 °C – stable male 25 °C | 0.27 (-0.08 – 0.65) |
| stable female 31 °C – fluctuating male 25 °C | **0.92 (0.56 – 1.33)** |
| fluctuating female 31 °C – stable female 25 °C | -0.18 (-0.63 – 0.25) |
| fluctuating female 31 °C – fluctuating female 25 °C | 0.06 (-0.14 – 0.29) |
| fluctuating female 31 °C – stable male 31 °C | **-0.66 (-1.1 – -0.22)** |
| fluctuating female 31 °C – fluctuating male 31 °C | -0.22 (-0.69 – 0.23) |
| fluctuating female 31 °C – stable male 25 °C | -0.35 (-0.78 – 0.1) |
| fluctuating female 31 °C – fluctuating male 25 °C | 0.31 (-0.16 – 0.73) |
| stable female 25 °C – fluctuating female 25 °C | 0.24 (-0.14 – 0.64) |
| stable female 25 °C – stable male 31 °C | **-0.48 (-0.84 – -0.12)** |
| stable female 25 °C – fluctuating male 31 °C | -0.05 (-0.47 – 0.36) |
| stable female 25 °C – stable male 25 °C | -0.17 (-0.53 – 0.2) |
| stable female 25 °C – fluctuating male 25 °C | **0.48 (0.09 – 0.85)** |
| fluctuating female 25 °C – stable male 31 °C | **-0.73 (-1.12 – -0.34)** |
| fluctuating female 25 °C – fluctuating male 31 °C | -0.28 (-0.72 – 0.13) |
| fluctuating female 25 °C – stable male 25 °C | **-0.41 (-0.82 – -0.04)** |
| fluctuating female 25 °C – fluctuating male 25 °C | 0.24 (-0.17 – 0.64) |
| stable male 31 °C – fluctuating male 31 °C | **0.44 (0.03 – 0.83)** |
| stable male 31 °C – stable male 25 °C | **0.31 (0.11 – 0.52)** |
| stable male 31 °C – fluctuating male 25 °C | **0.97 (0.58 – 1.33)** |
| fluctuating male 31 °C – stable male 25 °C | -0.13 (-0.53 – 0.27) |
| fluctuating male 31 °C – fluctuating male 25 °C | **0.53 (0.32 – 0.74)** |
| stable male 25 °C – fluctuating male 25 °C | **0.65 (0.27 – 1.03)** |

Supplementary Table S3. Back-transformed posterior expectations (in seconds; grouping factors including treatment, temperature, and sex) of conglobation time behavioural type and behavioural predictability of *Armadillidium vulgare*. Estimates and 85% credible (posterior probability) intervals are based on double hierarchical mixed models (reaction norm method).

|  | estimate (sec) |
| --- | --- |
| *behavioural type* |  |
| stable male 25 °C | 47.3 (32.1 – 66.0) |
| stable male 31 °C | 72.9 (49.5 – 101.0) |
| fluctuating male 25 °C | 19.2 (12.7 – 27.0) |
| fluctuating male 31 °C | 40.1 (25.1 – 59.0) |
| stable female 25 °C | 37.2 (24.9 – 51.7) |
| stable female 31 °C | 69.0 (45.7 – 96.8) |
| fluctuating female 25 °C | 26.9 (17.0 – 38.9) |
| fluctuating female 31 °C | 30.0 (17.2 – 46.3) |
| *behavioural predictability* |  |
| male 25 °C | 2.85 (2.45 – 3.32) |
| male 31 °C | 3.04 (2.59 – 3.57) |
| female 25 °C | 2.62 (2.28 – 3.02) |
| female 31 °C | 3.33 (2.81 – 3.94) |

Fig. S2. Back-transformed posterior distribution of temperature effect in population-level a) mean conglobation time (i.e., behavioural type) and b) residual intra-individual variation (rIIV) of conglobation time (i.e., behavioural predictability) in *Armadillidium vulgare* across sexes and treatment groups.

Figure S1. Effect of time on population-level mean conglobation time (i.e., behavioural type) in *Armadillidium vulgare*. Shaded areas represent 85% credible (posterior probability) intervals. Conglobation time is a latency variable; hence, lower values indicate higher risk-taking.

Supplementary Table S4. Estimates and 85% credible (posterior probability) intervals (in parentheses) of fixed and random effects (mean model) and residual standard deviation (dispersion model) of conglobation time in female and male *Armadillidium vulgare*. Estimates are based on double hierarchical mixed models (character state method). Estimates which were different from 0 based on their 85% credible intervals are bolded (note: variance estimates cannot overlap with zero as they are positively bound). *A. vulgare* in the stable group at 31 °C are set as reference group.

|  | model | | model | estimate (85% CI) |  |
| --- | --- | --- | --- | --- | --- |
|  |  | |  | females | males |
|  | *Mean model* | | *Fixed effects* |  |  |
|  |  | | Intercept | **0.33 (0.08 – 0.58)** | **0.43 (0.13 – 0.73)** |
|  |  | | Time | **0.09 (0.02 – 0.15)** | **0.09 (0.02 – 0.15)** |
|  |  | | Size | **0.26 (0.12 – 0.4)** | 0.09 (-0.14 – 0.34) |
|  |  | | Treatment |  |  |
|  |  | | fluctuating | **-0.63 (--1.12 – -0.16)** | **-0.49 (-0.93 – -0.05)** |
|  |  | | Temperature |  |  |
|  |  | | 25 °C | **-0.48 (-0.75 – -0.21)** | -0.29 (-0.62 – 0.03) |
|  |  | | Time of day |  |  |
|  |  | | pm | 0.1 (-0.03 – 0.23) | 0.09 (-0.04 – 0.23) |
|  |  | | Treatment × temperature | 0.43 (-0.02 – 0.88) | -0.24 (-0.67 – 0.19) |
|  |  | | *Random effects* |  |  |
|  |  | | stable treatment |  |  |
|  |  | | sd_intercept.ID_ 25 °C | **0.55 (0.36 – 0.8)** | **0.48 (0.23 – 0.77)** |
|  |  | | sd_intercept.ID_ 31 °C | **0.41 (0.21 – 0.65)** | **0.62 (0.37 – 0.94)** |
|  |  | | *r*_intercept.ID 25 °C – intercept 31 °C_ | **0.63 (0.21 – 0.92)** | 0.37 (-0.15 – 0.78) |
|  |  | | *r*_intercept.ID 31 °C – σ.ID 31 °C_ | -0.39 (-0.86 – 0.25) | -0.11 (-0.73 – 0.59) |
|  |  | | *r*_intercept.ID 31 °C – σ.ID 25 °C_ | -0.01 (-0.62 – 0.61) | -0.12 (-0.68 – 0.48) |
|  |  | | *r*_intercept.ID 25 °C – σ.ID 31 °C_ | -0.38 (-0.85 – 0.23) | -0.04 (-068 – 0.61) |
|  |  | | *r*_intercept.ID 25 °C – σ.ID 25 °C_ | -0.04 (-0.65 – 0.57) | 0.27 (-0.39 – 0.8) |
|  |  | | fluctuating treatment |  |  |
|  |  | | sd_intercept.ID_ 25 °C | **0.6 (0.35 – 0.91)** | **0.49 (0.27 – 0.75)** |
|  |  | | sd_intercept.ID_ 31 °C | **0.87 (0.56 – 1.26)** | **0.72 (0.48 – 1.02)** |
|  |  | | *r*_intercept.ID 25 °C – intercept 31 °C_ | **0.67 (0.28 – 0.93)** | **0.7 (0.34 – 0.94)** |
|  |  | | *r*_intercept.ID 31 °C – σ.ID 31 °C_ | -0.03 (-0.64 – 0.59) | 0.07 (-0.55 – 0.66) |
|  |  | | *r*_intercept.ID 31 °C – σ.ID 25 °C_ | 0.01 (-0.59 – 0.62) | 0.12 (-0.47 – 0.67) |
|  |  | | *r*_intercept.ID 25 °C – σ.ID 31 °C_ | 0.03 (-0.62 – 0.65) | 0.02 (-0.57 – 0.63) |
|  |  | | *r*_intercept.ID 25 °C – σ.ID 25 °C_ | -0.01 (-0.64 – 0.63) | 0.26 (-0.38 – 0.78) |
|  | Dispersion model | | *Fixed effects* |  |  |
|  | | Intercept | | **-0.18 (-0.36 – -0.003)** | **-0.25 (-0.39 – -0.09)** |
|  | | Size | | **-0.11 (-0.18 – -0.04)** | -0.09 (-0.21 – 0.02) |
|  | | Treatment | |  |  |
|  | | fluctuating | | -0.15 (-0.39 – 0.11) | -0.11 (-0.34 – 0.12) |
|  | | Temperature | |  |  |
|  | | 25 °C | | -0.23 (-0.48 – 0.01) | -0.07 (-0.31 – 0.15) |
|  | | Treatment × temperature | | 0.14 (-0.23 – 0.51) | 0.22 (-0.11 – 0.56) |
|  |  | | *Random effects* |  |  |
|  |  | | stable treatment |  |  |
|  |  | | σ.ID 31 °C | **0.24 (0.04 – 0.49)** | **0.12 (0.01 – 0.27)** |
|  |  | | σ.ID 25 °C | **0.21 (0.03 – 0.45)** | **0.23 (0.04 – 0.45)** |
|  |  | | *r*_σ.ID 31 °C – σ.ID 25 °C_ | 0.01 (-0.63 – 0.65) | 0.04 (-0.63 – 0.69) |
|  |  | | fluctuating treatment |  |  |
|  |  | | σ.ID 31 °C | **0.18 (0.02 – 0.41)** | **0.21 (0.03 – 0.45)** |
|  |  | | σ.ID 25 °C | **0.25 (0.03 – 0.53)** | **0.23 (0.03 – 0.5)** |
|  |  | | *r*_σ.ID 31 °C – σ.ID 25 °C_ | 0.09 (-0.59 – 0.73) | -0.06 (-0.68 – 0.6) |

Supplementary Table S5. Differences in adjusted repeatability (ΔR), among- (ΔV_w_) and within-individual variation (ΔV_i_) in conglobation time of *Armadillidium vulgare* between sexes, treatment groups and temperatures. Estimates are based on double hierarchical mixed models (character state method). Estimates which were different from 0 based on their 85% credible (posterior probability) intervals are bolded.

| contrast | ΔV_i_  mean (85% CI) | ΔV_w_  mean (85% CI) | ΔR  mean (85% CI) |
| --- | --- | --- | --- |
| stable male 31 °C – stable male 25 °C | 0.17 (-0.26 – 0.57) | 0.08 (-0.17 – 0.35) | 0.11 (-0.15 – 0.36) |
| fluctuating male 31 °C – fluctuating male 25 °C | 0.29 (-0.09 – 0.65) | -0.18 (-0.45 – 0.11) | **0.23 (0.003 – 0.46)** |
| fluctuating male 25 °C – stable male 25 °C | -0.008 (-0.41 – 0.38) | -0.14 (-0.44 – 0.16) | 0.001 (-0.32 – 0.29) |
| fluctuating male 31 °C – fluctuating male 31 °C | -0.13 (-0.69 – 0.47) | 0.12 (-0.14 – 0.38) | -0.12 (-0.41 – 0.19) |
| stable female 31 °C – stable female 25 °C | -0.14 (-0.44 – 0.14) | 0.27 (-0.02 – 0.56) | -0.19 (-0.45 – 0.02) |
| fluctuating female 31 °C – fluctuating female 25 °C | 0.41 (-0.22 – 0.98) | 0.09 (-0.18 – 0.35) | 0.13 (-0.15 – 0.39) |
| fluctuating female 25 °C – stable female 25 °C | 0.07 (-0.37 – 0.52) | -0.001 (-0.26 – 0.22) | 0.04 (-0.26 – 0.37) |
| fluctuating female 31 °C – fluctuating female 31 °C | 0.63 (-0.01 – 1.21) | -0.18 (-0.5 – 0.14) | **0.36 (0.11 – 0.64)** |
| stable male 31 °C – stable female 31 °C | 0.24 (-0.19 – 0.63) | -0.11 (-0.43 – 0.21) | 0.17 (-0.1 – 0.45) |
| stable male 25 °C – stable female 25 °C | -0.07 (-0.43 – 0.34) | 0.09 (-0.16 – 0.33) | -0.13 (-0.42 – 0.17) |
| variable male 31 °C – variable female 31 °C | -0.26 (-1.01 – 0.51) | -0.04 (-0.29 – 0.22) | -0.07 (-0.35 – 0.21) |
| variable male 25 °C – variable female 25 °C | -0.13 (-0.55 – 0.33) | 0.23 (-0.07 – 0.52) | -0.17 (-0.48 – 0.14) |
| stable male 31 °C – variable female 31 °C | -0.39 (-1.11 – 0.39) | 0.07 (-0.19 – 0.35) | -0.19 (-0.49 – 0.11) |
| stable male 31 °C – variable female 25 °C | 0.02 (-0.53 – 0.54) | 0.17 (-0.08 – 0.43) | -0.06 (-0.38 – 0.26) |
| stable male 25 °C – variable female 25 °C | -0.14 (-0.58 – 0.33) | 0.08 (-0.16 – 0.35) | -0.17 (-0.5 – 0.14) |
| stable male 25 °C – variable female 31 °C | -0.55 (-1.17 – 0.15) | -0.002 (-0.28 – 0.27) | **-0.29 (-0.6 – -0.02)** |
| variable male 31 °C – stable female 31 °C | 0.37 (-0.11 – 0.78) | -0.22 (-0.51 – 0.11) | **0.29 (0.05 – 0.56)** |
| variable male 31 °C – stable female 25 °C | 0.23 (-0.28 – 0.71) | 0.05 (-0.17 – 0.29) | 0.09 (-0.19 – 0.38) |
| variable male 25 °C – stable female 25 °C | -0.06 (-0.41 – 0.32) | 0.23 (-0.05 – 0.5) | -0.13 (-0.42 – 0.14) |
| variable male 25 °C – stable female 31 °C | 0.08 (-0.24 – 0.38) | -0.04 (-0.39 – 0.31) | 0.07 (-0.18 – 0.33) |

Fig. S3. Differences in adjusted repeatability (ΔR), among- (ΔVw) and within-individual variation (ΔVi) in conglobation time of females and males of *Armadillidium vulgare* between a) different temperatures in the same treatment group (fluctuating treatment group: variable; stable treatment group: stable), and between b) different treatment groups at the same temperature. Whiskers of varying thickness represent the 50% and 85% highest posterior density (HPD) intervals. Slopes not including zero are considered to have strong posterior support.
